# Annotations

**Published:** 1907-08-10

**Authors:** 


### ANNOTATIONS.

### The Term "Consultant."

In a previous issue we discussed the attempt which is being made in certain quarters to attach a definite and official significance to the term "consultant." The proposal is that this term shall be applied to practitioners who only see patients in conjunction with another practitioner, and who do not engage in the continuous care of patients. Whatever view may be taken of this proposal so far as the principle it embodies is concerned, it may be well to take note of the fact that the word "consultant" is not subject to any legal restrictions. Such terms as physician, surgeon, and medical practitioner are, of course, in an entirely different position. They have a legal basis and enjoy legal recognition; and their employment is confined to duly qualified members of the medical profession. But "consultant" is a term which anyone may adopt, and, as a matter of fact, it is sometimes adopted by those who practise medicine or surgery without any legal qualification. It is only necessary, for example, to glance at the advertisement columns of some of the most important of our lay contemporaries to gain an introduction to the "leading West-End consultant" on diseases of the hair. In these circumstances it may reasonably be asked whether the medical profession is well advised to select and dignify a term which may be adopted by anyone who chooses to use it. To the public one consultant will seem very much the same as another; and no one can deny that the unqualified pretender has as much right to the title as the qualified practitioner. For our own part, we have no sympathy with an attempt either arbitrarily to define the scope of any practitioner's work or to multiply professional distinctions. But, if the movement above referred to is to prevail, consulting physician or consulting surgeon is both a more correct and a safer title than "consultant."

### The Ventilation of Factories.

The Report of the Committee appointed by the Home Office to inquire into the best methods of securing the efficient ventilation of factories has just been issued. It is a document of great interest and of considerable value, and the adoption of the suggestions of the committee by employers will certainly tend to promote the health and longevity of the workers. Having in an interim report, issued in 1902, dealt with the general subject of ventilation and with the relation of an adequate supply of fresh air to the prevention of disease, the committee now proceeds to the consideration of the influence of dust and fumes introduced into the atmosphere of factories as the result of manufacturing processes. The principle for meeting this position should, in the view of the committee, be "that, wherever possible, dust, fumes and evil-smelling vapours should be dealt with at their point of origin, and never allowed to mix with the general atmosphere of a room." The application of this principle to various manufacturing conditions has been made the subject of a large number of experiments and observations, and in this practical part of the Report will be found valuable help to all those anxious to secure the protection and health of their workpeople. More especially does this statement apply to those industries which are known officially as "dangerous trades." Thus, in the match-making industry, repeated trials have at last secured a method by which the workers can be protected from all fume-contaminated atmosphere. Similarly, in dust-producing industries, as, for example, in the manufacture of knitting-needles and other bone articles, and in the carding of horse-hair removed from railway cushions, it has been possible to secure a perfectly clear atmosphere for the workers. These results, it must be noted, have been demonstrated as practically possible on the large scale; they are not merely the product of exceptionally favourable circumstances.

### Tobacco and the Profession.

In the circumstances of the time it was hardly to be expected that the annual meeting of the British Medical Association would escape the usual organised attempt to attach to the moderate use of alcohol the ban of professional opinion. But it is a somewhat new departure to find that it is proposed also to place tobacco under the same condemnation. This proposal appeared in a discussion conducted in the section of State Medicine, and some of the arguments advanced both on one side and the other lend force to the suggestion that the acquisition of technical knowledge and the possession of a medical diploma afford no guarantee of a logical and judicial habit of mind. The leader of the attack on the practice of tobacco smoking had convinced himself that the habit was founded entirely on sentiment and imitation. The boy thinks it a manly thing to imitate his elders, and continues the practice as he grows up because custom and association have bound him in fetters. Tobacco contains a deadly poison; how, then, can our people expect to enjoy good health when they consume three million pounds of it each year? For these stupid and limited reasons the nation, it seems, is engaged in a slow process of self-destruction. Another speaker announced that he had found tuberculosis more common in smokers than in non-smokers—a valuable observation were the numbers of the two classes equal. On the other hand, a third doctor argued that as tobacco smoke was destructive to many small insects and germs its use must be of value as a protective against infective disease. Here, again, was a suggestive claim, the force of which, however, seemed to disappear when the President remarked that to kill the bacillus of tubercle, tobacco must be used in so concentrated a form that it would at one and the same time kill the smoker. The presentation of a series of personal prepossessions, tastes and prejudices under the guise of a scientific discussion is hardly calculated to add to the popular appreciation of professional opinion. Apparently many of the speakers on these occasions have yet to learn that the definition of a merely personal creed is of no value to the public, however interesting it may be to the family circle of the orator.

